# Robust design from systems physics

**DOI:** 10.1038/s41598-020-70980-5

**Published:** 2020-08-31

**Authors:** Andrei A. Klishin, Alec Kirkley, David J. Singer, Greg van Anders

**Affiliations:** 1 Department of Physics, University of Michigan, Ann Arbor, MI 48109 USA; 2 Center for the Study of Complex Systems, University of Michigan, Ann Arbor, MI 48109 USA; 3 John A. Paulson School of Engineering and Applied Sciences, Harvard University, Cambridge, MA 02138 USA; 4 Naval Architecture and Marine Engineering, University of Michigan, Ann Arbor, MI 48109 USA; 5 Department of Physics, Engineering Physics, and Astronomy, Queen’s University, Kingston, ON K7L 3N6 Canada

**Keywords:** Statistical physics, thermodynamics and nonlinear dynamics, Statistical physics

## Abstract

A crucial challenge in engineering modern, integrated systems is to produce robust designs. However, quantifying the robustness of a design is less straightforward than quantifying the robustness of products. For products, in particular engineering materials, intuitive, plain language terms of strong versus weak and brittle versus ductile take on precise, quantitative meaning in terms of stress–strain relationships. Here, we show that a “systems physics” framing of integrated system design produces stress–strain relationships in design space. From these stress–strain relationships, we find that both the mathematical and intuitive notions of strong versus weak and brittle versus directly characterize the robustness of designs. We use this to show that the relative robustness of designs against changes in problem objectives has a simple graphical representation. This graphical representation, and its underlying stress–strain foundation, provide new metrics that can be applied to classes of designs to assess robustness from feature- to system-level.

## Introduction

**Figure 1:**
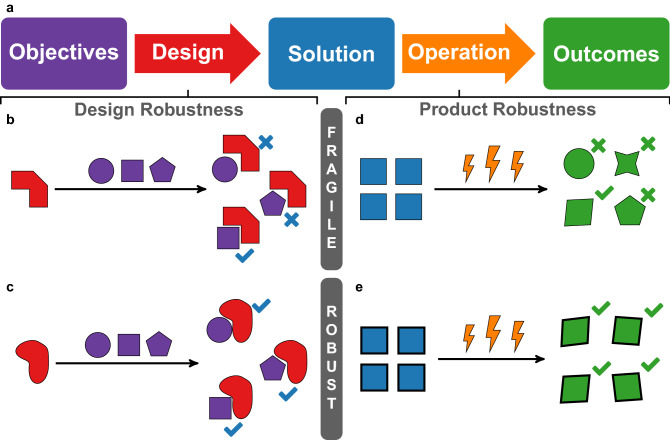
Schematic representation of the difference between a robust design and a robust product. (**a**) The pathway of product design and operation can be represented as a flow from a set of objectives, to a design process that produces a solution or product, followed by the testing and operation of the product results in one or more outcomes. The robustness of the product describes the product’s performance under different operating conditions. Robust products (**e**, blue squares) perform well under different operating conditions (lightning bolts), whereas “fragile” products (**d**) perform poorly. An analogous classification can also be applied to the design process. A robust design (**c**) is one in which the same solution (red shape) would be produced to meet different sets of objectives (purple shapes), whereas a fragile design (panel b) would not stand up to changes in the objectives.

**Figure 2:**
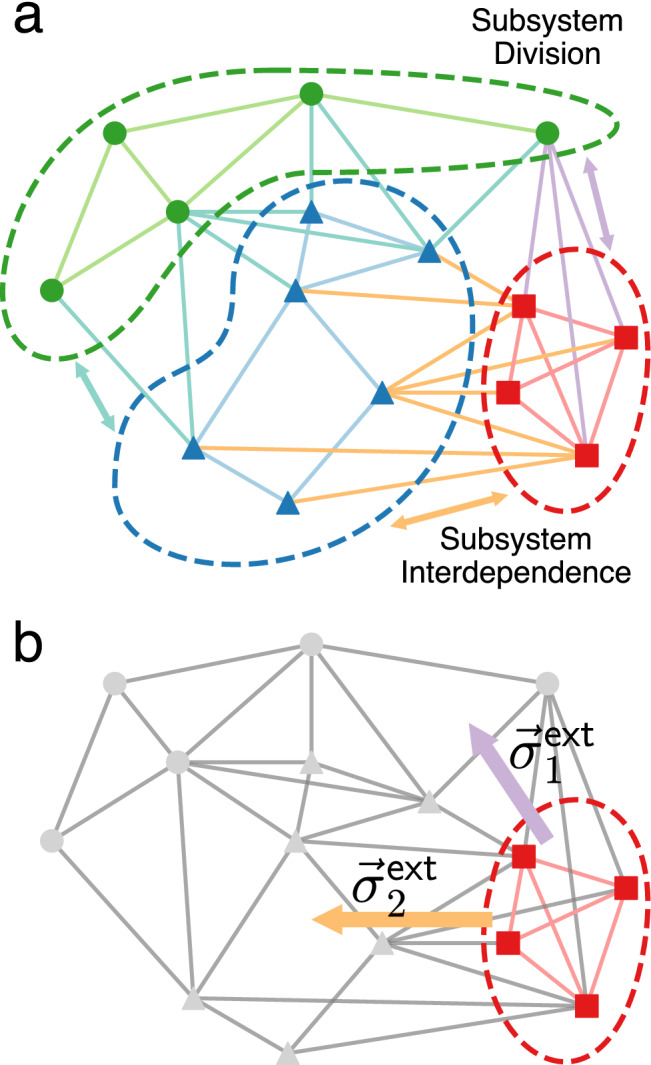
Schematic representation of interdependencies in a complex, integrated system. (**a**) Complex systems can be comprised of a set of connected, interdependent subsystems (distinguished by color). (**b**) A common design problem is to determine the relationship of a component subsystem (in red) to the other subsystems (gray). External subsystems induce “design stress” ($\vec {\sigma }^\mathbf{ext} _{1,2}$) on a subsystem that we use to characterize robustness.

Modern manufacturing and industrial development demand both robust products and robust designs. Whereas robust products exhibit similar, predictable behavior in a variety of operating conditions, robust designs preserve design elements under uncertainty in problem statements (see Fig. 1)^1,2^. Achieving robust design enhances supply chain stability, avoids rework, and thus reduces downstream cost and performance uncertainty^3,4^. Minimizing these uncertainties through robust design has become both increasingly important, and increasingly difficult to achieve, as products coming to market incorporate broader arrays of functionality that rely on the integration of heterogeneous subsystems^5^. The coupling of heterogeneous subsystems restricts subsystem component specifications, and small changes in the design of one subsystem can trigger avalanches of change in connected subsystems^6^. Preventing or controlling avalanches requires developing the ability to not only describe subsystem interdependencies, but also how interdependencies affect the robustness of subsystem and overall design (see Fig. 2).

Throughout engineering, the design of system elements often exploits known physical phenomena, leveraging knowledge developed through decades or centuries of investigation of basic physical science principles. For example, the principles of robustness of engineering materials have a long history and a rich language and mathematical apparatus. In this language, intuitive contrasts such as “brittle” versus “ductile” or “strong” versus “weak” achieve precise meaning in terms of performance thresholds on stress, or localized force, and strain, or localized displacement^7^. Unlike the study of materials, the study of the basic physical phenomena that underlie the behavior of systems integration is in its relative infancy^8–10^. Because of this relative infancy, what it means to be robust, and how to quantify robustness are open questions.

Here, we operationalize questions about the robustness of subsystem design via the “Systems Physics” approach of Ref.^11^. Via Systems Physics we draw quantitative comparisons between *design* classes, or architectures, at intermediate, “mesoscale” levels of analysis. We find that at the mesoscale, the robustness of architecture classes can be rigorously discussed in precisely the same terms that are used to quantify the robustness of materials, i.e., in terms of stress–strain relationships. By focusing on stress and strain thresholds, we classify the mesoscale designs by their response to external subsystem coupling as “brittle” or “ductile” and “strong” or “weak”. We show that the stress–strain analysis can be concisely summarized in two-factor robustness plots that directly compare system architectures. For concreteness, we show explicit examples of brittle, ductile, strong, and weak designs that arise in the context of a naval architecture-inspired arrangement problem. The design stress–strain curves, in the distributed systems we study, generically exhibit strain softening, i.e. a decrease in design stress with increasing design strain. We show that local architecture classes can change between brittle and ductile behavior depending on the form of global design pressure. This analysis of local manifestations of global design drivers provides a novel form of insight into a ubiquitous set of challenges faced in industrial design, as well as a new means of communicating about and achieving robust design.

## Robust design from statistical physics

Engineering complex systems is a difficult, longstanding problem. Early systematic design paradigms prescribed optimizing subsystems sequentially and independently, in the hope of forming a design “spiral” that narrows to a final, single solution^12^. These “point-based” design approaches rely heavily on mathematical optimization^13^. Optimal solutions, however, are only as good as the underlying models that produce them, and a key source of model uncertainty is the interaction of the model with external subsystems. The difficulty of design lies not in the individual subsystems but in their integration^14^. Large systems further compound the potential for integration failure^15^. The failure potential can be mitigated by focusing not on finding “good” solutions, but by focusing on avoiding “bad” ones. Broad-based, so-called “Set-Based Design”, paradigms have become influential across automotive^16,17^, aerospace^18^, and naval design^19^. Regardless of the domain, a key challenge in Set-Based Design is to comb through a space of potential design solutions and eliminate ones that have elements that are likely to lead to future problems. Those future design problems are likely to arise when design elements are not robust.

However, describing robustness in set-bassed and other flexible design paradigms requires new approaches. Robustness approaches in narrowing, convergent design^20,21^ describe single-design solutions, living at a point in design space. In contrast, set-based design paradigms require determining the robustness not of *a* design, but of *sets* of designs. The quantitative, collective treatment of sets is precisely the subject of statistical physics.

Statistical physics has a long history of describing collective behaviors that range from the long-known thermodynamics of gases^22^, to more recent investigations of entropy-driven order^23–26^, and a host of non-thermal collective phenomena, including flocking behaviors^27^, the collective motion of human crowds^28^, traffic jams^29^, the synchronization of agricultural yields^30^, and primate social dynamics^31^. A central advantage of statistical physics is its ability to group together microscopic system states and investigate the properties of and transitions between those groups in the language of free energy (see the conceptual and mathematical discussion below). Free energy ideas have been used to describe collective behavior in biological swarms^32^ and and in brain activity^33^. Work has also shown^11^ that statistical physics approaches can be applied to systems design problems, under the guise of Systems Physics. Here, we use Systems Physics to study the intermediate-scale structure of design spaces to study design robustness.

### General approach

To establish a physics approach for understanding robust systems design, we take cues from the physics of materials. In materials an instructive example is a steel rod under mechanical load. Under load the rod can take one of two qualitatively different states, intact or broken. Before it breaks, the response of a rod to external forcing can be quantified using material-dependent relationships between force and deformation, i.e. stress–strain relationships. The nature of the stress–strain response of the rod can be used to concretely describe its material along the independent axes of *weak–strong* and *brittle–ductile*^7^. Brittle and ductile materials show qualitative differences in behavior. Both brittle and ductile behavior can, in different industrial contexts, find appropriate uses. But in either case, determining which material to use requires knowing how it behaves.

Adapting the materials analogy to systems design requires identifying the key behaviors and what drives them. In systems design a key factor in robustness is how design elements behave as they integrate with other subsystems (see schematic illustration in Fig. 2a). Their behavior, in terms of how specifications, locations, etc., respond to integration is driven by multiple factors. Different subsystems are generally designed by different designers to satisfy different *design objectives*. Also generally, each design objective has different relative importance, which we term *design pressure*. Design pressures act on all the elements at the same time and thus represent an externally imposed, whole-system level, or *global* drive.

Global design pressures manifest themselves *locally* by driving specific design elements in different directions, e.g. in terms of their physical locations or specifications. For example, competing design pressures of cost and performance can produce discord in the specification of design elements. This discord at the level of elements or subsystems, is analogous to local mechanical stresses and strains that occur in materials under external load. This suggests there should be an analogous local measures of force and deformation, i.e. *design stress* and *design strain*, that express “locally” how design elements respond to the “load” of global design objectives.

**Figure 3:**
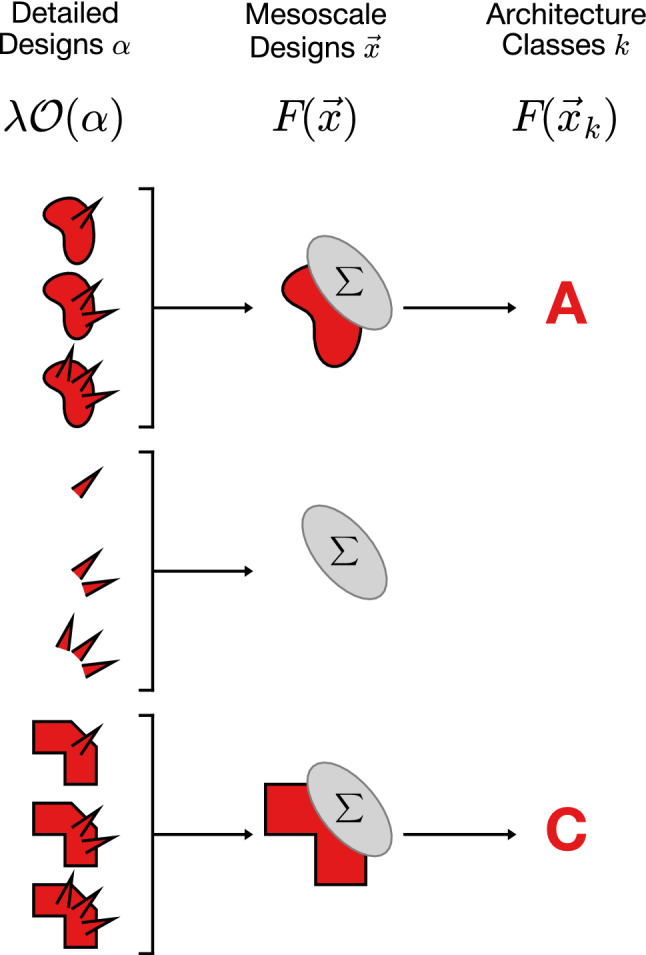
Detailed designs $\alpha$ group into mesoscale designs $\vec {x}$, and locally optimal mesoscale designs form architecture classes *k*. Each detailed design is characterized by multiple features (here system shape and spike pattern) and quantified by the design objective $\lambda {{\mathscr {O}}}(\alpha )$. We use the system shape as the feature $\vec {x}$ to define mesoscale designs by summing over all spike patterns to get the Landau free energy $F(\vec {x})$. Top and bottom shapes are each better than the middle one and thus form architecture classes, here *A* and *C*, in the local free energy minima $F(\vec {x}_k)$.

If global design pressures are connected to local design stress and strain, how can this be quantified? The challenge in quantifying the global–local connection is that meeting global design objectives is the collective result of all component elements. Moreover, when design features could be placed in a number of possible locations and/or could meet different specifications, this produces a combinatorial explosion of possible design states. The challenge of describing the collective behaviors of combinatorially large numbers of states has an analogue in the thermodynamics of atomic systems, a problem that prompted the development of statistical physics. Here, instead of using it to group states of atoms, we will use statistical physics to group designs.

Our statistical physics approach to quantify design stress and strain relies on grouping designs that share at least one feature. (see Fig. 3 for an illustration). We term a group of designs with a shared feature as a *mesoscale* design. Each mesoscale design needs to be described by a quantity that encodes its properties. The first quantity that needs to be encoded is the number of designs the mesoscale design has grouped by common feature. Mesoscale designs that group many detailed designs need to be distinguished from ones that group few. The second quantity that needs to be encoded is how well the grouped designs meet global design objectives. These two factors can be lumped together into a mathematical function, referred to as a *free energy* in statistical physics. The free energy of a mesoscale design decreases as the number of detailed designs that comprise it increases, or as the detailed designs better satisfy the design objectives, or both. In contrast, changes that reduce the number of detailed designs or their suitability for the objectives increase the free energy.

By grouping designs and computing their free energy, mesoscale designs reduce the complexity of a large number of detailed designs to be reduced to the consideration of a small number of features of interest. Describing designs in terms of features of interest has two advantages. First, features of interest that sit at local minima of the free energy correspond to locally optimal mesoscale designs. The deviation from a locally optimal feature set gives a definition of *design strain* and the free energy change this deviation induces can be used to define *design stress*. Second, The set of designs that receive a “pull” in design stress toward the same feature set, akin to a watershed, constitute a “basin” in design space that can define an *architecture class*.

Grouping designs by feature sets provides each architecture class with a definition of design stress and strain. Appropriate stress and strain definitions, in turn, inform a two-factor determination of robustness. To see why, the materials analogy is again instructive. In materials, robustness is determined by response to mechanical stress and strain. Mechanical stress and strain give two key performance indicators of material performance under different kinds of external influence: maximal loading, or “ultimate stress”, and maximum deformation, or “ultimate strain”. In everyday language, materials with high ultimate stress are strong and low ultimate stress are weak; materials with low ultimate strain are brittle and high ultimate strain are ductile. Given design analogues of material stress and strain, computing the analogous robustness stress and strain thresholds will provide weak/strong and brittle/ductile classifications for designs.

**Figure 4:**
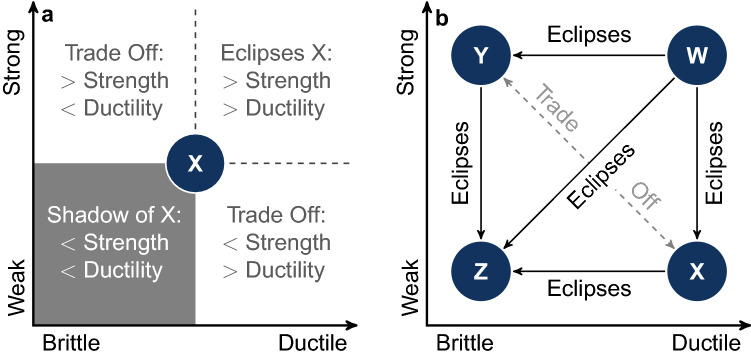
Two-factor robustness ($R^2$) comparison of design architectures. Design architectures can be described by their response to external design stress (e.g., changes in cost) or design strain (e.g., changes in specification limits). The stress–strain response can be used to determine the robustness of an architecture, and to compare architectures. (**a**) shows that locating an architecture on robustness axes of weak–strong and brittle–ductile facilitates the comparison of that architecture ${\mathsf {X}}$ with other potential architectures. The existence of architecture ${\mathsf {X}}$ casts a “shadow” (lower left quadrant) over other potential architectures that would be less robust by both measures (lower strength, lower ductility). Conversely situating ${\mathsf {X}}$ also identifies (upper right quadrant) forms of robustness, that if they were found in other potential architectures would “eclipse” the robustness of ${\mathsf {X}}$ (greater strength, greater ductility). The other two quadrants (upper left, lower right) describe regions where potential architectures would involve a trade-off in forms of robustness (either in strength or ductility) between architectures. (**b**) illustrates how this could inform comparison of a set of architectures ${\mathsf {W}}$, ${\mathsf {X}}$, ${\mathsf {Y}}$, and ${\mathsf {Z}}$. In this illustration ${\mathsf {W}}$ eclipses all other architectures either in terms of strength (${\mathsf {X}}$) ductility (${\mathsf {Y}}$) or both (${\mathsf {Z}}$). If architecture ${\mathsf {W}}$ did not exist, ${\mathsf {Z}}$ is eclipsed by both ${\mathsf {X}}$ (in ductility) and ${\mathsf {Y}}$ (in strength) but a choice between ${\mathsf {X}}$ and ${\mathsf {Y}}$ means a trade-off between the two forms of robustness.

Knowing how weak/strong or brittle/ductile particular design architecture is is useful. However, it is also important to compare the robustness of designs. To make this comparison, the existence of weak/strong and brittle/ ductile contrasts suggests plotting architectures on two axes that run weak–strong and brittle–ductile. A sketch of this is given in Fig. 4a. An architecture ${\mathsf {X}}$ can be located on a pair of axes representing the two measures of robustness. The region around ${\mathsf {X}}$ can be divided into quadrants. Additional architectures would fall into one of those quadrants, permitting a direct comparison of robustness. We refer to the lower left quadrant as the shadow of ${\mathsf {X}}$ because architectures falling in that region would be inferior to ${\mathsf {X}}$ in both strength and ductility. In contrast ${\mathsf {X}}$ would be in the shadow of any architecture that falls in the upper right quadrant, because that architecture would have greater strength and ductility. We refer to that region as the eclipsing region of ${\mathsf {X}}$. The other two quadrants, in the upper left and lower right, are regions where architectures would involve trade-offs with ${\mathsf {X}}$, either greater strength but reduced ductility, or greater ductility but reduced strength.

An example robustness comparison between is sketched in Fig. 4b. Figure 4b gives an example of a two-factor robustness plot ($R^2$-plot) for four architectures, which we label ${\mathsf {W}}$, ${\mathsf {X}}$, ${\mathsf {Y}}$, and ${\mathsf {Z}}$. In the scenario depicted in the $R^2$-plot 4b, ${\mathsf {W}}$ has the same ductility as ${\mathsf {X}}$, but ${\mathsf {W}}$ is stronger, so all else being equal the architecture ${\mathsf {W}}$ would represent a more robust choice. Similarly, architectures ${\mathsf {W}}$ and ${\mathsf {Y}}$ have the same strength, but ${\mathsf {W}}$ is more ductile, so all else being equal ${\mathsf {W}}$ would represent a more robust choice. Similar reasoning also indicates that ${\mathsf {W}}$ is more robust than ${\mathsf {Z}}$ in both strength and ductility. If architecture ${\mathsf {W}}$ was unavailable, similar considerations would make ${\mathsf {Z}}$ a less robust choice than either ${\mathsf {X}}$ or ${\mathsf {Y}}$. Comparing ${\mathsf {X}}$ and ${\mathsf {Y}}$ shows that the two architectures have different forms of robustness, ${\mathsf {X}}$ is more ductile, but ${\mathsf {Y}}$ is stronger. In this case, all else being equal, the designer would need additional information to determine whether strength or ductility is likely to be the more important measure of robustness.

Figure 4 illustrates what could occur in a design process under fixed external design pressure. However, changes in design pressure can change the robustness of architectures, echoing e.g. the brittle–ductile transitions that occur in industrial materials^34^ and geology^35^. Similar to the varied industrial uses of both brittle and ductile materials, we anticipate the usefulness of architecture classes manifesting different forms of robustness. In evaluating robustness, our goal is to generate knowledge about the possible emergent behaviors in the design space to inform the human designer, who would make the final design choice.

### Systems physics

In this section we cast the foregoing general approach into a concrete mathematical form. Our mathematical model expands upon Systems Physics, a statistical mechanics framework for design problems introduced in Ref.^11^. We consider a design problem with a combinatorially large ensemble of candidate *detailed design* solutions $\{\alpha \}$. For each detailed design $\alpha$ we compute several quantitative *design objectives* ${{\mathscr {O}}}_i$, where *i* is an index. An example of a numerical design objective would be the cost of routing a cable between two functional units; we explain the design objectives for our model system in the next section. Given a set of design objectives, a standard calculational device from statistical mechanics that is applied in analogous problems is to associate an expected average outcome $\left\langle {{\mathscr {O}}}_i\right\rangle$ with each objective. Given only the above data, information theory implies that the minimally biased (maximal entropy^36^) probability distribution for choosing a detailed design $\alpha$ that matches the objectives with their outcomes is given by

1 $$\begin{aligned} p(\alpha )=\frac{1}{{{\mathscr {Z}}}}e^{-\sum \limits _{i}\lambda _i {{\mathscr {O}}}_i(\alpha )}, \end{aligned}$$

where ${{\mathscr {Z}}}$ is a normalization constant. High probability designs are the ones that best fulfill the competing design objectives. Whereas the design objectives ${{\mathscr {O}}}_i$ assumed to be known *a priori* and represent *what* criteria need to be considered by the designer, the design pressures $\lambda _i$ represent *how much* each criterion matters, and the choice of these pressures could differ between stakeholders with different concerns, e.g. cost versus performance. Selecting particular values of $\lambda _i$ significantly shapes the probability distribution $p(\alpha )$ and can radically and abruptly change the types of the preferred designs $\alpha$. Predicting and quantifying the preferred designs within the ensemble is a goal of Systems Physics^11^.

The properties of the whole ensemble are contained in the normalization of Eq. (1) that can be computed as

2 $$\begin{aligned} {{\mathscr {Z}}}=\sum _{\alpha }e^{-\sum \limits _{i}\lambda _i {{\mathscr {O}}}_i(\alpha )}, \end{aligned}$$

and has the familiar form of a partition function from statistical physics. In statistical physics, the partition function encodes the statistical averages of design objectives across the whole ensemble^11^. However, summing over the whole ensemble masks the fact the many detailed designs $\alpha$ achieve the same design objective value ${{\mathscr {O}}}_i$, and thus obscures the intermediate scale design drivers. Discovering these design drivers requires studying designs at a higher level of granularity. This granularity is given by the architecture classes we discussed in general terms above, and which we will now define more precisely.

### Architecture classes

We achieve a higher level of granularity by selecting a design feature $\vec {x}_\alpha$ that multiple detailed designs $\alpha$ share. An example of a shared feature could be the spatial location of a particular functional unit or one of its internal operational parameters (e.g. pressure or voltage). Regardless of the specific feature chosen, a set of designs sharing a common feature $\vec {x}$ can be described by a *mesoscale design* (see Fig. 3). The feature space $\{\vec {x}\}$ is typically much smaller than the set of detailed designs $\{\alpha \}$, but can be used to recover the statistical information of the full design ensemble via the expression

3 $$\begin{aligned} {{\mathscr {Z}}}=\sum _{\vec {x}} e^{-F(\vec {x})}, \end{aligned}$$

where $F(\vec {x})$ is the *free energy*. The free energy quantifies the effective design objective of the mesoscale design $\vec {x}$, and is determined by the expression

4 $$\begin{aligned} e^{-F(\vec {x})}=\sum \limits _{\alpha }\delta (\vec {x}-\vec {x}_\alpha ) e^{-\sum \limits _{i}\lambda _i {{\mathscr {O}}}_i(\alpha )}. \end{aligned}$$

Here $\delta (\vec {x}-\vec {x}_\alpha )$ is an indicator function, equal to 1 when the detailed design $\alpha$ belongs to the mesoscale design $\vec {x}$ and 0 otherwise. $F(\vec {x})$ is an example of a so-called Landau free energy^37^, and provides a mesoscale characterization of classes of designs that share characteristics specified by $\vec {x}$. The procedure of going from a large ensemble of detailed designs $\alpha$ to mesoscale designs $\vec {x}$ is known as *coarse graining* in statistical physics^37^. Coarse graining applied to a model system and the resulting free energy landscape are illustrated in Fig. 5.

The shape of the free energy landscape *F* indicates the relative preference between different mesoscale designs. Designs that accord better with the balance of design pressures $\lambda _i$ have smaller *F*, and vice versa. Local minima of $F(\vec {x})$ are “best-in-class” designs. We denote these designs as $\vec {x}_k$, and index them as $k\in \{A,B,C,\ldots \}$ (colored circles in Fig. 5b). However, although best-in-class designs are defined to be those that best meet a fixed set of design objectives, changes in the specification of the objectives can alter the classification, so understanding the robustness of designs is crucial.

### Quantifying robustness

A deviation from a local minimum $\vec {x}_k$ within the feature space gives a design strain $\vec {\epsilon }_k=\vec {x}-\vec {x}_k$. In design strain coordinates, design stress is given by the free energy gradient $\vec {\sigma }(\vec {\epsilon }_k)=-\vec {\nabla }F(\vec {x}_k+\vec {\epsilon }_k)$. Sufficiently close to the local minimum, design stress pulls the design back to the minimum, i.e. $\vec {\sigma }\cdot \vec {\epsilon }_k<0$. However, at larger strains in a particular direction, the design can reach a threshold, or saddle point, in free energy and get pulled by the local design stress to a different minimum. We call that point the *ultimate strain* and formally define it as

5 $$\begin{aligned} \vec {\epsilon }_k^{\;{\textsf {ult}}}= \mathop {\mathrm{arg\,min}}\limits _{\vec {\epsilon }_k} \left| \vec {\epsilon }_k\right| :\; \vec {\sigma }\cdot {\hat{\epsilon }}_k>0, \end{aligned}$$

where $|\cdot |$ denotes a suitable vector norm (here we use standard Euclidean norm) and ${\hat{\epsilon }}_k$ is a unit vector pointing along the strain direction. The operator ${{\,\mathrm{arg min}\,}}$ finds the closest saddle point but still returns the vector $\vec {\epsilon }_k^{\;{\textsf {ult}}}$ rather than just its norm. We illustrate the path from local free energy minima to the saddle points in an example system in Fig. 6a,c,e.

As a mesoscale design is strained from $\vec {0}$ to $\vec {\epsilon }_k^{\;{\textsf {ult}}}$, it will develop design stress. To analyze the stress response, it is convenient to compute the projection of the stress along the strain direction, $\sigma =\left| \vec {\sigma }(\vec {\epsilon }_k)\cdot {\hat{\epsilon }}_k\right|$. From this projection it is possible to compute the *ultimate stress*, i.e. the magnitude of externally exerted stress that causes designs to switch between classes. Formally, this is given by

6 $$\begin{aligned} \sigma _k^{\textsf {ult}}=\max \limits _{a\in [0,1]} \left| \vec {\sigma }(\vec {x}_k +a \vec {\epsilon }_k^{\;{\textsf {ult}}})\cdot {\hat{\epsilon }}_k^{\textsf {ult}}\right| , \end{aligned}$$

where *a* is an auxiliary variable parametrizing a straight line from $\vec {0}$ to $\vec {\epsilon }_k^{\;{\textsf {ult}}}$. While the free energy minimum $\vec {x}_k$ defines the “best-in-class” mesoscale design, the basin of all mesoscale designs that design stress brings back to the best-in-class design defines an *architecture class*.

We have defined architecture classes thus far for isolated subsystems. When subsystem designers incorporate effects that arise from coupling to other subsystems, other subsystems exert *external* design stress or strain on the subsystem of interest. External stress and strain correspond, respectively, to what are referred to in statistical mechanics as “intensive” (size independent) or “extensive” (size dependent) modifications of the specification of the system.

sGoing back to the analogy of a metal rod, this nomenclature reflects that a mechanical load can be applied to the rod with two protocols. One is to subject the rod to a fixed external stress force, or intensive modification, and measure the resulting strain. The other is to subject the rod to a fixed linear strain, or extensive modification, in form of stretching or compression and measure the resulting stress.

Protocols for materials response have direct analogues in systems design. In systems design, an example of external design stress would arise from the need to route a connection from a functional unit to an external subsystem, with the direction and cost per unit length specified for the connection. This scenario creates a uniform design stress $\vec {\sigma }^{\textsf {ext}}$ on the subsystem (Fig. 5a), and the new local optimum would be found at the location where the internal design stress balances the external $\vec {\sigma }^{\textsf {ext}}+\vec {\sigma }=0$. An example of external strain would be the need to position an additional object in the location $\vec {x}_k$, thus requiring the shift of subsystem design features by design strain of $\vec {\epsilon }{\,}^{\textsf {ext}}$ away from the minimum. In either case, external stress or strain may or may not push the mesoscale design into a different architecture class basin. Resisting the architecture class shift is the property that we call *robustness*.

We determine the robustness of each architecture class by computing the design stress–strain curves. From these curves, we extract the ultimate stress and strain for each architecture class and plot them together without averaging in Fig. 8. These stress–strain relationships facilitate the characterization of each design architecture class as weak or strong by comparing the respective $\sigma _k^{\textsf {ult}}$ among different architecture classes *k*. Weak designs have small $\sigma _k^{\textsf {ult}}$, whereas strong designs have large $\sigma _k^{\textsf {ult}}$. We also characterize designs as brittle or ductile by comparing the relative $|\vec {\epsilon }_k^{\;{\textsf {ult}}}|$. Brittle designs have small $|\vec {\epsilon }_k^{\;{\textsf {ult}}}|$, whereas ductile designs have large $|\vec {\epsilon }_k^{\;{\textsf {ult}}}|$. Considering both strength and ductility gives us the two-factor robustness, $R^2$, of the architecture classes, presented in Fig. 9.

### Beyond ultimate stress

Whereas the determination of ultimate strain can characterize the robustness of an architecture class, it is also important to understand what happens once architecture classes are pushed beyond their viability limit. Doing so requires understanding the configuration of architecture classes under large external stress.

To model the external stress, we consider not only the free energy of the subsystem of interest $F(\vec {x})$ that depends on the design feature $\vec {x}$, but also the free energy of an external subsystem $F'(\vec {x}\,')$ that depends only on the design features $\vec {x}\,'$ of the external subsystem. The interdependence of the two subsystems is captured by the interaction free energy $F_{int}(\vec {x},\vec {x}\,')$. The goal is to understanding what happens in the system of interest, under the assumption the external subsystem can take any configuration it prefers. We carry this out by integrating out the external subsystem, leaving a description of the remaining subsystem of interest. Mathematically, the procedure is similar to to the earlier coarse-graining procedure in Eq. (4),

7 $$\begin{aligned} e^{-{\tilde{F}}(\vec {x})}=\sum \limits _{\vec {x}\,'} e^{-F(\vec {x})-F'(\vec {x}\,')-F_{int}(\vec {x},\vec {x}\,')}. \end{aligned}$$

In general, performing the computation in Eq. (7) is challenging, since it requires a detailed model of the external subsystem. However, we note that free energy is only defined up to an additive constant which does not affect the locations or properties of local minima. Thus we can get insight into the effect of external couplings by adopting a simplified form of the interaction free energy:

8 $$\begin{aligned} F_{int}(\vec {x},\vec {x}\,')\approx (\vec {x}\,'-\vec {x})\cdot \vec {\sigma }^{\textsf {ext}}+\text {const.} \end{aligned}$$

This form of interaction free energy describes a uniform external design stress $\vec {\sigma }^{\textsf {ext}}$ applied to each feasible design in the domain of design feature $\vec {x}$, caused for example by the addition of a cable of fixed direction and cost per unit length. Computationally, this form of interaction makes the summation in Eq. (7) separable and gives the effective free energy landscape as:

9 $$\begin{aligned} {\tilde{F}}(\vec {x})=F(\vec {x})-\vec {x}\cdot \vec {\sigma }^{\textsf {ext}}+\text {const.} \end{aligned}$$

In general, ${\tilde{F}}$ and *F* will have different sets of local minima, i.e. design configurations that are best-in-class will change in the presence of external stress. We investigate the effect of variable external stress by considering different vectors $\vec {\sigma }^{\textsf {ext}}$ that span the space $\{\vec {\sigma }^{\textsf {ext}}\}$. If the external stress $\vec {\sigma }^{\textsf {ext}}$ is much larger than any internal design stresses $-\vec {\nabla }F(\vec {x})$ naturally arising in a given architecture class, the subsystem is completely dominated by external stress that eliminates candidate architecture classes, reducing design richness.

We characterize the loss of design richness under external stress by finding the domain in $\{\vec {\sigma }^{\textsf {ext}}\}$ space in which a minimum of the same type *k* exists. Here, by “same” we mean a minimum that moved less than some threshold $\Delta x^{\textsf {th}}$ under a small change of stress $\delta \vec {\sigma }^{\textsf {ext}}$

10 $$\begin{aligned} \left| \vec {x}_k(\vec {\sigma }^{\textsf {ext}}+\delta \vec { \sigma }^{\textsf {ext}})-\vec {x}_k(\vec {\sigma }^{\textsf {ext}})\right| <\Delta x^{\textsf {th}}\;. \end{aligned}$$

We illustrate how external stress affects the viability ranges of subsystem design classes in Fig. 7 using Venn diagrams in the $\{\vec {\sigma }^{\textsf {ext}}\}$ space. Together, the viability ranges for architecture classes and the analysis of their ultimate design stress and strain constitute the quantitative knowledge required for robust system design.

## Example system: model, results, and discussion

**Figure 5:**
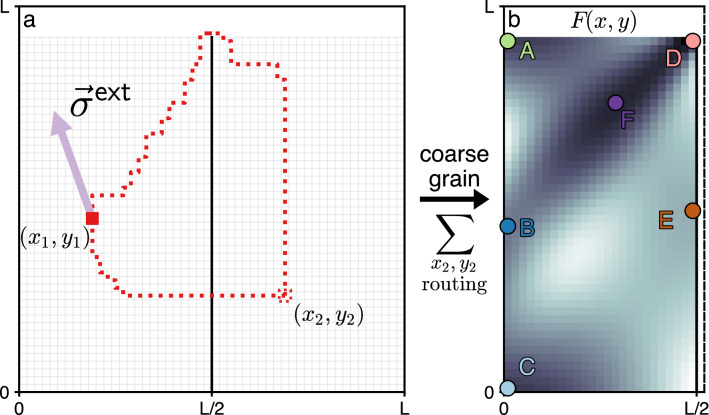
Schematic representation of the specific subsystem investigated for robustness. (**a**) Two connected functional units are placed in positions $(x_1,y_1)$ and $(x_2,y_2)$. There are two qualitative ways to connect them along the shortest Manhattan route: either directly by drilling a hole through the bulkhead, or by first routing up to the bulkhead, over it and back down. The position of the first unit is taken as the design feature $\vec {x}$, while the position of the second one and the possible routings are “integrated out” by computing the free energy via Eq. (13). The external design stress on the system has the form of a constant force $\vec {\sigma }^{\textsf {ext}}$ shown with a purple arrow. (**b**) Coarse graining procedure leads to the free energy landscape *F*(*x*, *y*) for the possible positions of the first unit in the part of the domain left of the bulkhead. Local free energy minima are identified with architecture classes labelled with capital letters *A* through *F* and distinct colors.

### Model system

The Systems Physics based robustness analysis developed in the previous section can be applied to a broad range of design problems. For concreteness, we will illustrate its use in arrangement problems that arise in Naval Architecture, taking as a specific example an established model of early stage ship design^6^. Our ship design model involves embedding a network of many functional units into a ship hull of fixed geometry, and routing connections between the units. Large-scale effects on unit routing induced by changes in design pressure were studied in Ref.^11^. Here, we study this model at the mesoscale to understand the robustness of system design. To determine robustness, we focus on a subsystem with two connected functional units that is externally connected to other subsystems, modelled via external design stress (see Fig. 5a).

The subsystem is situated in a square domain of $L\times L$ discrete cells. The domain is separated along the middle line into two compartments housing one functional unit each, divided up to height $h_{\textsf {bh}}$ by a watertight bulkhead. The bulkhead serves to prevent simultaneous water flooding of both compartments in case one of them is breached. It is possible to drill a hole through the bulkhead, reinforce that hole, and route a connection through it. However, such a hole bears risks that affect ship survivability. To assess how survivability considerations affect the routing problem, we consider two distinct types of routing: either along the shortest possible route through the bulkhead, or along the shortest possible route around the top of the bulkhead. The connections are only routed horizontally and vertically, so there is a large but finite number of possible routings for each choice of positions of the two units. A particular realization of unit positions and routings of two types is shown in Fig. 5a. The possible positions of the two units $(x_1,y_1)$ and $(x_2,y_2)$ along with the choices of particular routing form the detailed design space $\{\alpha \} = \{(x_1,y_1,x_2,y_2,\text {routing})\}$.

Within the design space, detailed designs are evaluated with respect to two design objectives, ${{\mathscr {O}}}_1,{{\mathscr {O}}}_2$, and corresponding design pressures $\lambda _1,\lambda _2$

11 $$\begin{aligned} {{\mathscr {O}}}_1\equiv&E=C\left( |\Delta x|+|\Delta y| \right) ,&\lambda _1&\equiv 1/T; \nonumber \\ {{\mathscr {O}}}_2\equiv&B,&\lambda _2&\equiv \gamma . \end{aligned}$$

The first design objective *E* represents the cost of the routing, linearly proportional to the Manhattan (taxicab/grid) length of the routing used. The corresponding design pressure is *inverse cost tolerance* *T*, similar to the thermodynamic temperature. Low cost tolerance means that designs with shorter routings are strongly preferred, whereas high cost tolerance means that cost is not a strong factor in choosing a design. The second design objective $B\in \{0,1\}$ is a binary variable indicating whether a given design routes through the bulkhead (1) or goes around (0). The corresponding design pressure is the bulkhead penalty $\gamma$ that quantifies the survivability penalty associated with routing through the bulkhead. Low, near-zero, values of $\gamma$ mean that routing through or around the bulkhead are equally preferable, whereas high values of $\gamma$ strongly suppress routing through the bulkhead.

In terms of these specific design objectives and design pressures, the partition function (2) takes the form

12 $$\begin{aligned} {{\mathscr {Z}}}=\sum _{\alpha }e^{-\frac{E(\alpha )}{T}-\gamma B(\alpha )}, \end{aligned}$$

where the sum over $\alpha$ runs over *the whole set* of possible detailed designs. To group the detailed designs into mesoscale designs, we use the position of the *left* functional unit $\vec {x}=(x_1,y_1)$ as the design feature of interest. The position of the right functional unit $(x_2,y_2)$ and the routings are integrated out. The position of the left unit then has the associated free energy *F*(*x*, *y*)

13 $$\begin{aligned} e^{-F(x,y)}=\sum \limits _{\alpha }\delta (\vec {x}-\vec {x}_1(\alpha ))e^{-\frac{E(\alpha )}{T}- \gamma B(\alpha )}. \end{aligned}$$

An example of the free energy landscape is shown in Fig. 5b. Local minima of the free energy are associated with the architecture classes *A* through *F*. We find that the free energy landscape and the pattern of architecture classes vary greatly with the design pressures $T,\gamma$, and that in all cases the design robustness is given directly by Eqs. (5) and (6). There are several large-scale reorganizations between architecture classes as the design pressures *T* and $\gamma$ are varied. These reorganizations are analogous to phase transitions in thermodynamic systems, but are not sharp transitions because the design problems we study have finite size. Despite the finite sizes, it is possible to approximately determine where the reorganization of architectures occurs^11^. In this model reorganization occurs around a *cost phase transition*: at low $T<T_{\textsf {crit}}\sim C/\ln 2$ units prefer short connections to minimize the routing cost, whereas at large $T>T_{\textsf {crit}}$ they prefer long connections to maximize the flexibility in carrying out the routing^11^. Another large-scale reorganization is the transition of the average bulkhead penetration $\left\langle B \right\rangle$, or fraction of designs routing through the bulkhead: it approaches 0 for simultaneously large *T* and $\gamma$ (preferring flexibility in routing and suppressing bulkhead penetration), and approaches 1 when either *T* or $\gamma$ is small (preferring low-cost routing and allowing bulkhead penetration, or a combination of both). As we will find below, the origin of these large-scale reorganizations can be traced in the mesoscale through the appearance and disappearance of architecture classes and changes in their robustness.

To capture the reorganization of architecture classes, we study the routing problem for a range of design pressures $\{T,\gamma \}$. We fix our system of units by setting *C* = 1.0, which fixes the units of cost tolerance *T*. To maximize illustrative power, we seek a set of choices of $\gamma$ and *T* that allows for the study of all the possible architectural organizations with the fewest number of state points. For this purpose, a suitable choice is to scan along the line of $\gamma =2.0$ with $T\in [0.5,2]$ because that choice crosses both the cost- and bulkhead penetration-based reorganizations. We also make specific choices of system geometry parameters. Since the positions of the functional units are discrete, we cannot reliably resolve the stress–strain curves on length scales less than 1 cell. Moreover, the comparison of robustness between architecture classes requires the system domain to be sufficiently large to support multiple architecture classes. To this end, we set the domain size *L* = 50, with the bulkhead going up to $h_{\textsf {bh}}=46$, though our analysis and results are similar for different system sizes. We find that ultimate strains vary from 1.5 to 18 cells, allowing us to reliably distinguish the architecture classes along both weak–strong and brittle–robust axes.

### Results and discussion

#### Design stress and strain

**Figure 6:**
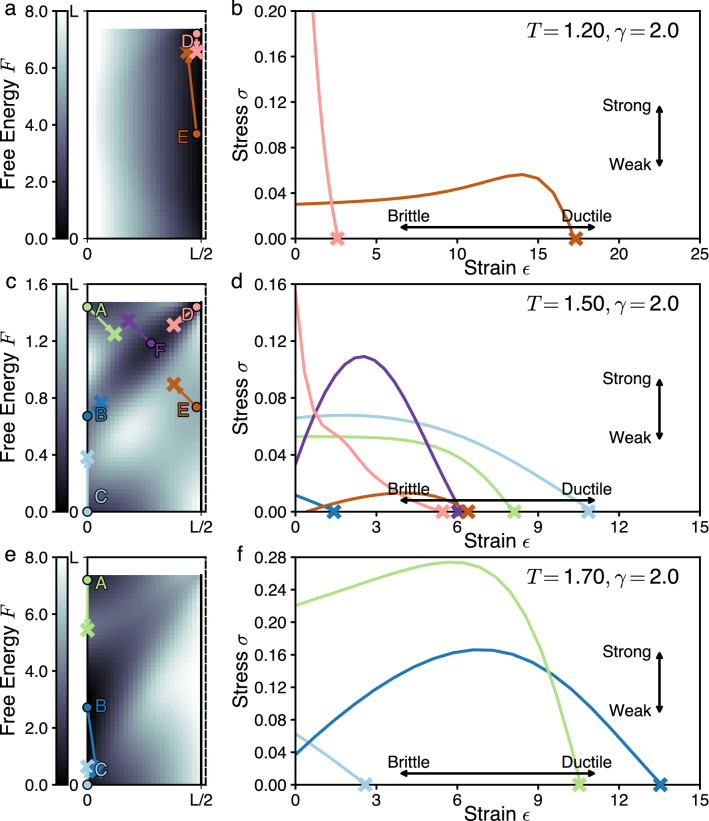
Statistical physics approach quantifies stress–strain relationships in design problems. Plots show free energy landscapes and stress–strain curves at three different cost tolerances *T* = 1.20, 1.50, 1.70 and constant bulkhead penalty $\gamma =2.0$. (**a**,**c**,**e**) Free energy landscape for the position of the left functional unit. The solid vertical line on the right denotes the position of the bulkhead. The dashed vertical line cuts off the domain of the second functional unit that has been integrated out. Note the different colormap scales at different *T*. Each colored circle indicates a local minimum that forms an architecture class, indexed with a unique letter *A* through *F* and a unique color (green, blue, purple etc.). The cross marks and lines connecting them to circles indicate the ultimate strain locations for each minima, as determined by condition (5). (**b**,**d**,**f**) Stress–strain curves for each of the local minima at given *T*, with stress measured along the ultimate strain direction via spline interpolation of the free energy landscape. The cross marks indicate the ultimate strain $\epsilon ^\text {ult}_k$ for each minimum. The maximum of each curve indicates the ultimate stress for each minimum $\sigma ^\text {ult}_k$.

**Figure 7:**
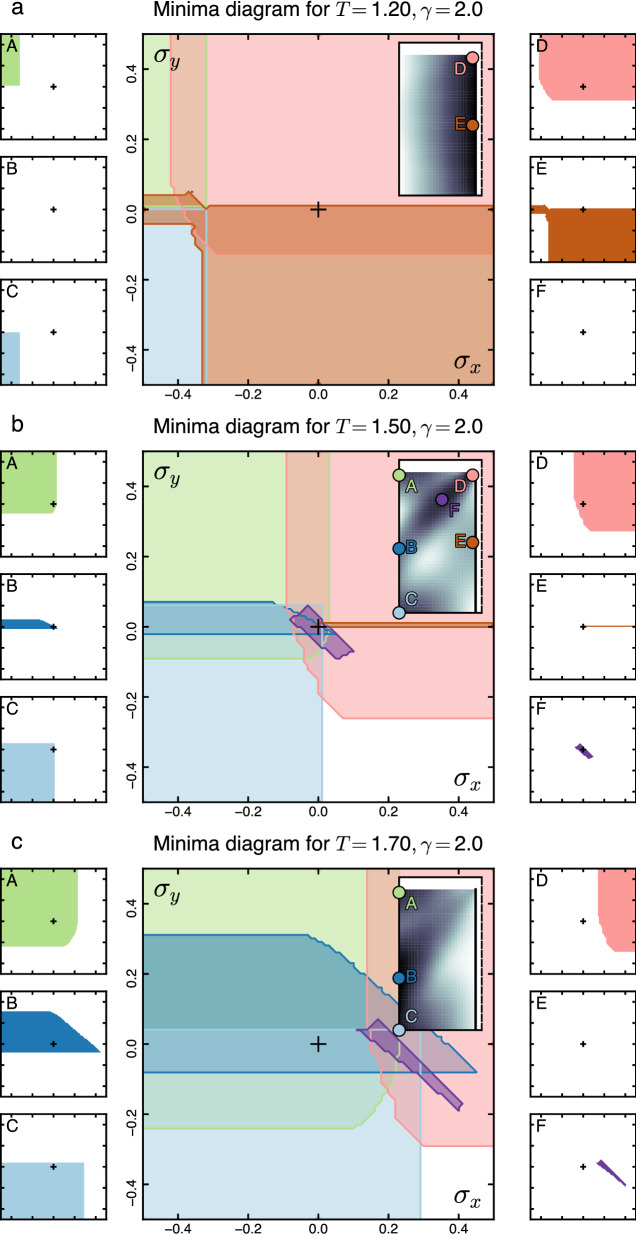
The response to external stress gives viability limits on architecture classes and shows external stress can become viable under external stress. Plots shows regions of existence of architecture classes in the $(\sigma ^{\textsf {ext}}_x,\sigma ^{\textsf {ext}}_y)$ (external stress) plane. (**a**) *T* = 1.20, (**b**) *T* = 1.50, (c) *T* = 1.70. For each panel, (center) Venn diagram of regions in the $(\sigma ^{\textsf {ext}}_x,\sigma ^{\textsf {ext}}_y)$ plane where each of 6 architecture classes exists. The black crosses indicates the origin of the plane $\vec {\sigma }^{\textsf {ext}}=0$. (inset) Free energy landscape with the architecture classes *A* through *F* labelled in color. (sides A–F) Regions in the $(\sigma ^{\textsf {ext}}_x,\sigma ^{\textsf {ext}}_y)$ plane where the corresponding individual minima exist, in the same stress plane as the central diagram.

Figure 6 demonstrates the range of architecture classes and their respective stress–strain curves that appear in the model subsystem. For this subsystem, for each fixed *T* in the range of [0.5, 2.0] we identify as many as 6 qualitatively different architecture classes that we label *A* through *F*. Figures 6 and 7 illustrate the architecture classes at three representative values of *T* = 1.20, 1.50, 1.70. *T* = 1.20 corresponds to the low-cost regime. *T* = 1.70 corresponds to the high flexibility regime. *T* = 1.50 corresponds to the regime in which there is a near balance between cost and flexibility.

At each cost tolerance, for each architecture class we compute the stress–strain response (Fig. 6b,d,f). As described above, for materials the stress–strain response can be measured via two principal protocols. In the first, the material is deformed by a fixed strain $\epsilon ^{\textsf {ext}}$ and equilibrates at some corresponding stress $\sigma (\epsilon ^{\textsf {ext}})$. In the second, the material is affected by a fixed stress $\sigma ^{\textsf {ext}}$ and equilibrates at some corresponding strain $\epsilon (\sigma ^{\textsf {ext}})$. Graphically, this is equivalent to picking first a point on the vertical $\sigma$ axis and finding the corresponding curve point on the horizontal $\epsilon$ axis, or vice versa.

#### Stress–strain: comparison with materials

For common materials, the difference in protocols is not very noticeable since at low stress and strain their relationship is linear, at larger deformations it is weakly nonlinear but still monotonic, up until the breaking point at finite stress and strain. However, this textbook materials science picture breaks down for the design stress–strain relationship in our example design problem in three important ways, at both low and high strain.

The differences between stress–strain relationships for designs and materials can is facilitated by expanding the relationships at low strain as a power series

14 $$\begin{aligned} \sigma =\sigma _0 +Y\epsilon +O(\epsilon ^2)\;. \end{aligned}$$

The first deviation from common material behavior is that for design stress the constant term is nonzero $\sigma _0\ne 0$ for all of the curves in Fig. 6b,d,f. Similar effects are common in manufactured engineering components that exhibit residual internal stress, usually resulting from plastic deformations in manufacturing, thermal expansion, boundary effects, or phase change of materials^38^. The main implication of residual stress is that applying small external design stress $\sigma ^{\textsf {ext}}<\sigma _0$ does not result in measurable design strain, as opposed to the conventional linear response.

The second deviation is that the linear part of stress response can be both positive ($Y>0$, as in Fig. 6f, architecture classes *A*, *B*, green and dark blue curves) and negative ($Y<0$, same figure, architecture class *C*, light blue). A positive linear response means that the architecture class can support at least small design stress above $\sigma _0$ level via a small deformation. A negative linear response means that the ultimate stress $\sigma ^{\textsf {ult}}=\sigma _0$ and is already reached for $\epsilon =0$, or no strain. For any higher, supercritical external stress, there is no corresponding point $\epsilon (\sigma ^{\textsf {ext}})$ and thus the architecture class immediately “breaks”, transitioning the design to a different class.

Whereas the first two deviations from textbook materials response are observed at low strain, the third one appears at high strain, right before the breaking point. Many conventional materials (e.g. steel) break at finite stress. However, some materials (e.g. fiber-reinforced brittle concrete^39^) exhibit a different phenomenon known as “tension softening”^40^, whereby they support decreasing amounts of stress as they are strained, and ultimately fail at zero stress. We observe this phenomenon in all of the architecture classes we find in the present model system.

Together, these three deviations describe the unconventional pathways in which architecture classes can break. Via strain: If the external subsystem coupling provides a fixed design strain, the design stress remains positive and finite for a wide strain range, ensuring that the chosen architecture class remains viable. Via stress: Conversely, if the external subsystem coupling provides a fixed stress, the architecture class only responds noticeably beyond a certain stress threshold, but often responds with an abrupt architecture class change. Via stress and strain: a combination of external design stress and strain can push the architecture class into the tension softening regime, making it unviable.

#### Beyond ultimate stress

Figure 6 illustrates what happens to an architecture class as it is strained up to its limit of viability by showing the design stress along the direction of least ultimate strain. This is useful for assessing the further viability of an architecture following the effects of an external design strain. However, it can also be important to determine the effects on the number of architecture classes under the influence of external design stress that can push one or more classes beyond their limit of viability.

To assess robustness in this form, Fig. 7 illustrates the effect of the external design stress taking any values in the plane $(\sigma ^{\textsf {ext}}_x,\sigma ^{\textsf {ext}}_y)$. In this plane, each architecture class *A* through *F* has a viable domain. We find that the shapes of these domains are complex, implying that the viability of an architecture class is highly sensitive to both direction and magnitude of external stress. We show the overlap of the domains of all six architecture classes in the center of each panel in Fig. 7 in form of a computed Venn diagram.

We start analysis with the richest Venn diagram at the near-critical *T* = 1.50 (Fig. 7b). In that diagram, the classes *E*, *F* (pink and purple) are viable in very narrow and specific ranges of external stress $(\sigma ^{\textsf {ext}}_x,\sigma ^{\textsf {ext}}_y)$. Compared with the other architectures, small amounts of uncertainty in external design stress would be be sufficient to render architecture classes *E*, *F* unviable. At the same time, the architecture classes *A* through *D*, in which the functional unit is localized either in one of the three corners or the middle of one side of the allowed domain, are viable given almost any amount of external design stress outwards toward the domain boundaries, as well as moderate stress directed inwards toward the middle of the domain. This form of analysis gives a more detailed understanding variations in the robustness of architecture classes when the effects of external design stress are not uniform in all directions.

From the depiction of the response to anisotropic stress in Fig. 7 it can be seen that external stress can, indeed, push some architectures beyond their viability limits while leaving others viable. However, Fig. 7 also shows that in situations where there is low cost tolerance (i.e., a strong preference for low cost, *T* = 1.20, panel a) or high cost tolerance (i.e., a weak preference for low cost, *T* = 1.70, panel c), the external stress can make viable some architecture classes that would not be viable without the external stress. Architectures *A* and *C* are examples of this at low cost tolerance (panel a), whereas architectures *D* and *F* are examples of this at high cost tolerance (panel c).

**Figure 8:**
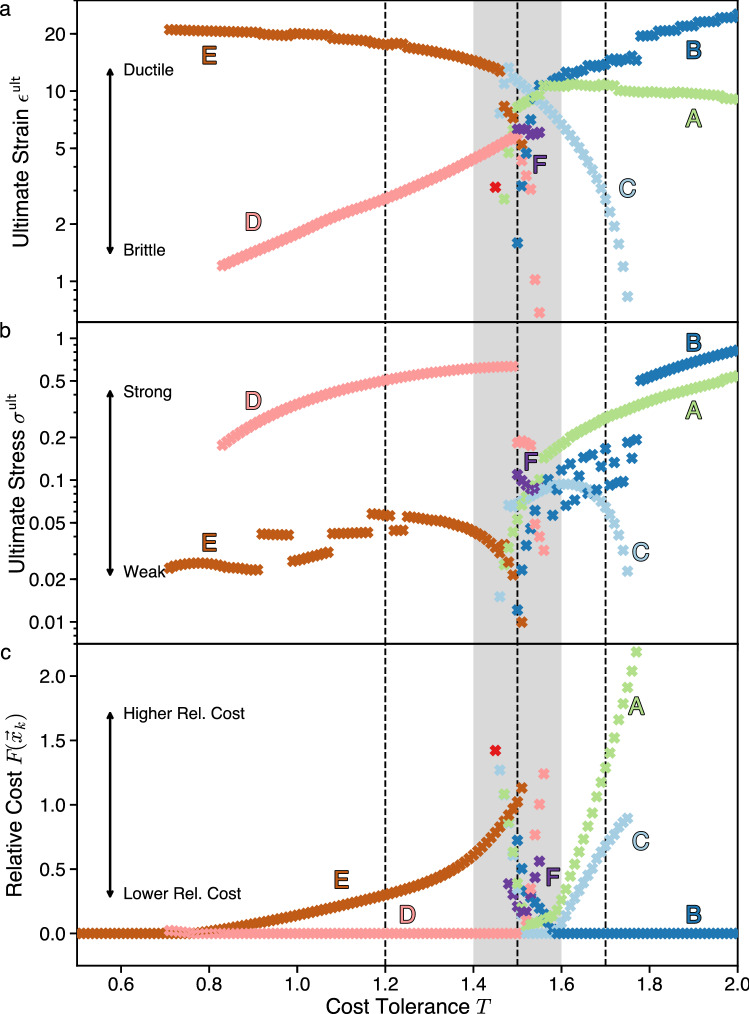
Architecture classes can transition strong to weak, or from brittle to ductile if design pressures change. Plots give robustness and optimality measures for all architecture classes (local minima of Landau free energy $F(\vec {x})$) existing at each value of cost tolerance *T*. The gray-shaded area on all three graphs indicates the region near $T_{\textsf {crit}}=1/\ln 2\approx 1.44$, which exhibits almost all of the architecture classes. The three dotted vertical lines indicate the values of *T* for a detailed analysis is given in Figs. 6 and 7. Color and letter coding remain the same as in those Figures. (**a**) Ultimate strain values, corresponding to the *brittle–ductile* robustness characterization of architecture classes. (**b**) Ultimate stress values, corresponding to the *weak–strong* robustness characterization of architecture classes. (**c**) Landau free energy $F(\vec {x}_k)$ values at all local minima, normalized so that for any *T* the lowest *F* value is zero. Vertical position of the points corresponds to *higher-lower relative cost* characterization of architecture classes.

#### Robustness and design pressure changes

Figures 6 and 7 showed a detailed analysis of how particular architecture classes respond to external design forcing at three representative values of cost tolerance *T*. To validate that these choices of *T* are representative, and to better understand how robustness changes in response to changes in design pressure, Fig. 8 aggregates the specific results shown in Figs. 6 and 7 with the results of similar analysis over a fine grid of $T\in [0.5,2]$. As we scan the *T* range, the robustness of architecture classes experiences a clear shift in the region of *T* where the architectures reorganize from low-cost to high flexibility. In the low-cost regime, architecture classes *D*, *E* are viable, and their properties are typified by the prior analysis at *T* = 1.20. In the high-flexibility regime, architecture classes *A*, *B*, *C* are viable, and their properties typified by the prior analysis at *T* = 1.70. At intermediate values of *T* where there is a reorganization between these regimes (shaded area in Fig. 8) we observe the highest diversity of viable architectures at zero stress. However, we also observe sharp changes in the robustness of architectures in response to external stress or strain. These change in robustness of the mesoscale design precisely in the near-critical *T* region suggests a causal relationship: the shift between viable architecture classes is the primary mechanism to drive the large-scale phase transitions in the whole design space.

**Figure 9:**
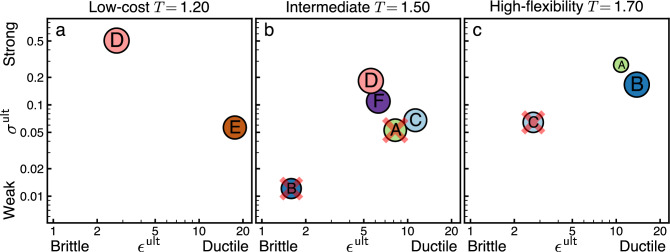
Two-factor robustness ($R^2$) comparison for model-system architecture classes facilitates the elimination of non-robust (weak/brittle) architectures. The analysis of the model system implements the scheme sketched in Fig. 4, using the two robustness factors of ultimate stress ($\epsilon ^{\textsf {ult}}$, vertical axis) and ultimate strain ($\sigma ^{\textsf {ult}}$, horizontal axis). The three panels depict the architectures and robustness relations among them at different cost tolerances *T* (*T* = 1.2-a, *T* = 1.5-b, *T* = 1.7-c). Architectures are represented by circles with size proportional to global design objectives. At high cost tolerance (**c**), where design pressure favors flexibility in realizing designs, the architecture C (also marked with a red $\times$) falls in the shadow of both architectures A and B. Between architectures A and B there is a trade-off in robustness between strength and ductility. A similar trade-off exists at low cost tolerance (**a**) between architectures D and E. At intermediate tolerance (**b**) where there is a balance of concern between cost and flexibility, more architectures are possible. Comparing their robustness, the architecture A is eclipsed by C, the architecture B falls in the shadow of all other architectures, and a trade-off exists between the three architectures C, D, and F.

#### Two-factor robustness comparison of architecture classes

The analysis above gave detailed information about the structure of the design space. Working from detailed information about the space, it is possible to distill essential pieces of information that can inform design decisions. Decisions involving comparing the robustness of architectures can be cast in the form of $R^2$-plots. Whereas the Fig. 4 diagrams were schematic illustrations, applying the analysis framework illustrated in Fig. 4 to our model system yields the $R^2$-plots shown in Fig. 9.

Figure 9 gives a proof-of-principle that two-factor comparisons of architectures can be successfully computed in model systems. However, for a given model system the comparisons themselves are interesting. First, in the model systems we study, we find no case in which a single architecture class eclipses all others in robustness in both strength and ductility in a $R^2$-plot. We find that, in the low-cost regime *T* = 1.20 (Fig. 9a), the trade-off is between the only two existing architecture classes: architecture *D* is stronger, but more brittle, whereas architecture *E* is weaker but more ductile. In the regime where design pressure favors designs that can be realized with more flexibility, *T* = 1.70 (Fig. 9c), there is a strength/ductility trade-off between architectures *A*, *B*, both of which eclipse architecture *C*. In the intermediate regime, where design pressures for cost and flexibility are nearly balanced, *T* = 1.50 (Fig. 9b), there is a three-way strength/ductility trade-off between architectures *C*, *D*, *F*, but both of their robustness factors are much smaller than at either higher or lower *T*. These results indicate that the comparison of robustness between architectures is complex. It depends both on the system being designed and on the design pressures, and that even simple models can exhibit trade-offs.

## Conclusion

In this paper we described a method to engineer systems that are robust to variation in the design problem statement. We situated robustness in design as a necessary complement to the robustness of a product under changes in the operating environment (Fig. 1). We showed that the robustness of a design is not a unitary concept, but instead it takes multiple forms including being described on the axes brittle–ductile and weak–strong (Figs. 4, 9). We found that these axes describe system design in the same mathematical terms that are used to describe the robustness of materials. The approach developed in this paper sets the stage for the further investigation of robust design.

First, to give a concrete demonstration of the application of our approach we used an example model system drawn from arrangement problems in Naval Engineering^6^. In that model system we found that the design architectures generically manifested forms of residual stress and tension softening that are observed in materials systems. Though both phenomena are well-characterized in materials, they are phenomena that are associated with special forms of preparation that introduce boundary effects (residual stress) or by unconventional micromechanics in the material (tension softening)^39^. Our observation of these effects in a model of system design raises the question of whether that observation is specific to our model system, or whether system designs behave, in general, like unconventional materials. The fact that the microscopic interactions in systems design can easily have more varied forms than the micromechanics of materials suggests that conventional behavior for systems may resemble unconventional behavior in materials, but further investigation is required.

Second, though we needed to show the approach on a concrete model system, it can be extended straightforwardly to other classes of design problems. We believe that for a broad class of problems, the two-factor classification of robustness ($R^2$-plot; Fig. 4) will provide a straightforward means for designers to compare the robustness of different design architecture classes. The comparison of classes in our example showed that the $R^2$ analysis provides a straightforward means to eliminate weak/brittle architecture classes. We expect this to be useful in complex system design where it has been argued that eliminating bad choices is more important than selecting good ones^19^. The nontrivial trade-offs in $R^2$ we found here suggest that a similar multifaceted characterization will be useful in broader classes of complex systems design.
