# Publisher Correction: Robust design from systems physics

**DOI:** 10.1038/s41598-020-75648-8

**Published:** 2020-10-30

**Authors:** Andrei A Klishin, Alec Kirkley, David J Singer, Greg van Anders

**Affiliations:** 1 Department of Physics, University of Michigan, Ann Arbor, MI 48109 USA; 2 Center for the Study of Complex Systems, University of Michigan, Ann Arbor, MI 48109 USA; 3 Paulson School of Engineering and Applied Sciences, John A, Harvard University, Cambridge, MA 02138 USA; 4 Naval Architecture and Marine Engineering, University of Michigan, Ann Arbor, MI 48109 USA; 5 Department of Physics, Engineering Physics, and Astronomy, Queen’s University, Kingston, ON K7L 3N6 Canada

Correction to: *Scientific Reports* 10.1038/s41598-020-70980-5, published online 31 August 2020

This Article contains errors in Figure 3, where the rotated "teardrop" shaped icons in the center row are missing due to a technical error. The correct Figure 3 appears below as Figure 1.

**Figure 1:**
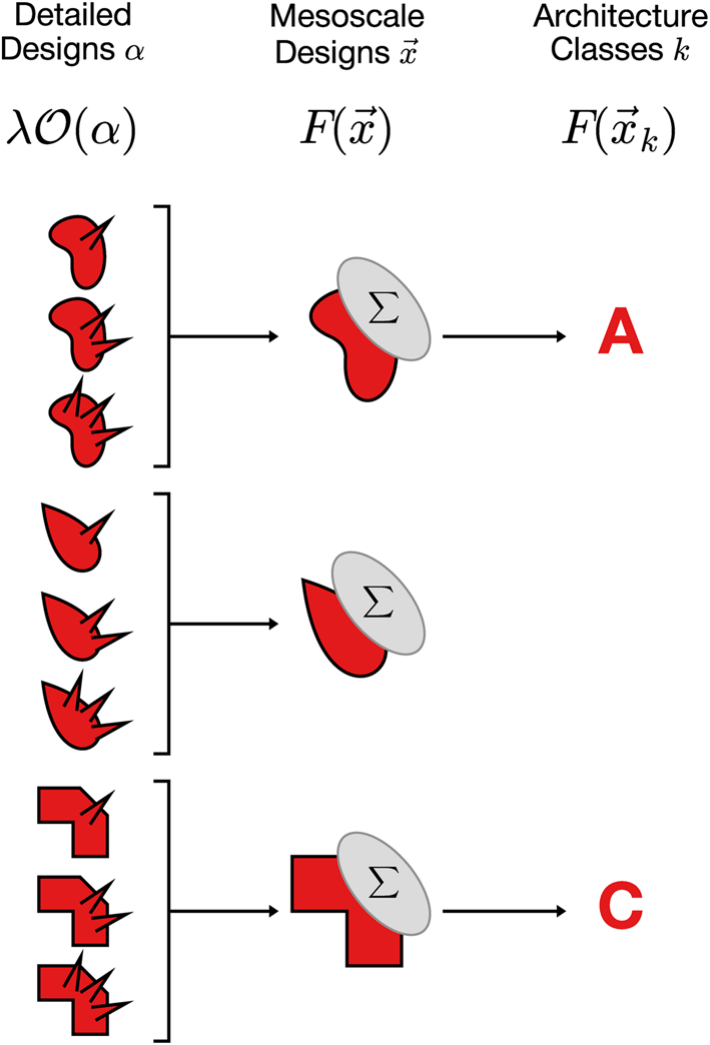
A correct version of the original Figure 3.

